# Psychometric analysis of the adult sickle cell quality of life measurement information system (ACSQ-Me) in a UK population

**DOI:** 10.1186/s12955-019-1136-7

**Published:** 2019-04-29

**Authors:** Owen Cooper, Hayley McBain, Sekayi Tangayi, Paul Telfer, Dimitris Tsitsikas, Anne Yardumian, Kathleen Mulligan

**Affiliations:** 1 School of Health Sciences, City, University of London, EC1V 0HB, London, UK; 2 East London NHS Foundation Trust, London, UK; 3 Barts Health NHS Trust, London, UK; 4 Homerton University Hospital NHS Foundation Trust, London, UK; 5 North Middlesex University Hospital, London, UK

**Keywords:** Sickle cell disease, Quality of life, ASCQ-Me, SF-36, Validity

## Abstract

**Background:**

The Adult Sickle Cell Quality of Life Measurement Information System (ASCQ-Me) has been shown to be a reliable and valid questionnaire measuring health-related quality of life (HRQoL) in the US sickle cell disease (SCD) population. The study objective was to test the validity and reliability of the ASCQ-Me for use in the UK.

**Methods:**

The US ASCQ-Me, Hospital Anxiety and Depression Scale (HADS), self-reported symptoms, and Medical Outcome Survey Short Form 36 (SF-36) were administered to 173 patients with SCD. Clinical severity was assessed by the number of painful episodes indicated by hospital admissions.

**Results:**

The results showed that the item banks of the UK ASCQ-Me had good internal consistency. Anxiety and depression were strongly correlated with the emotional, and social item banks of the UK ASCQ-Me, with moderate correlations between the UK ASCQ-Me item banks and SF-36 components suggesting convergent validity. A confirmatory factor analysis confirmed the conceptual framework of the scale as being the same as the US ASCQ-Me, indicating construct validity. Known groups validity was found, with the ASCQ-Me being able to differentiate by SCD severity groups.

**Conclusion:**

The analysis of the sample shows evidence of both validity and reliability of the ASCQ-Me for use in the UK SCD population.

**Electronic supplementary material:**

The online version of this article (10.1186/s12955-019-1136-7) contains supplementary material, which is available to authorized users.

## Background

Sickle cell disease (SCD) is an inherited structural haemoglobin disorder, common in people whose family origins were from Africa, but also seen in people with family origins in the Eastern Mediterranean, Middle East and South Asia. In England, it is now the most common serious inherited disorder, with a birth prevalence of approximately 1 in 2000 [1]. There are three primary genotypes of SCD: haemoglobin SS (HbSS); haemoglobin SC (HbSC); and haemoglobin Sβ-thalassæmia (HbSβThal). An estimated 12,500 to 15,000 people in the UK have SCD [2].

SCD is one of the most common reasons for hospital admission and has the highest rate of multiple admissions for individual patients in the UK [3]. SCD has been found to adversely affect health-related quality of life (HRQoL), but there are few studies that have evaluated SCD HRQoL in adults in Europe. In a sample of 96 adults with SCD, one study [4] found that HRQoL was significantly lower than that of the UK general population. To date, HRQoL has however, been assessed in adults with SCD using generic measures [5, 6], such as the RAND Medical Outcomes Study 36-item Short Form Survey (SF-36) [7] or EuroQol five-dimensional questionnaire (EQ-5D) [8]. Generic measures are required to enable comparison with other diseases as well as with the general population [9]. These measures, however, do have limitations as they do not measure the specific effects of the disease, and thus may not detect subtle, but clinically important variations in quality of life [10]. Disease-specific measures are likely to be more sensitive than generic measures to clinically significant change [11] as well as being more relevant to the disease under study [9].

HRQoL is an important outcome of clinical trials in SCD (Pecker et al., 2017), however there is currently no disease-specific HRQoL measure for adults with SCD that has been validated for use in the UK [6]. Two disease-specific HRQoL measures have been developed and validated in the US: the Sickle Cell Impact Measurement Scale (SIMS) [12], and the Adult Sickle Cell Quality of Life Measurement Information System (ASCQ-Me) [13, 14]. The SIMS was adapted from four existing questionnaires: the Arthritis Impact Measurement Scale (AIMS) [15] and three generic measures. It has four domains: pain; physical functioning; emotional well-being; social functioning. The SIMS validation study compared HRQoL in adults with SCD and rheumatoid arthritis (RA). No difference was found between the two patient groups on overall HRQoL but people with SCD scored higher than those with RA on physical and social domains [12]. The ASCQ-Me items were derived from research with adults who have SCD and their health care providers. It has been validated showing the item banks to be sensitive to SCD severity based on a self-reported medical history checklist, and validity has been shown utilising item response theory [16, 17]. It was found that the ASCQ-Me also had similar disciminant validity to the Patient-Reported Outcomes Measurement Information System (PROMIS) [18] in regards to SCD severity [17]. Physical function, pain, and the ability to engage in social roles and activities, as measured by the ASCQ-Me, were most affected by SCD severity. All ASCQ-Me validation studies to date have been conducted in US samples. It is necessary to validate patient reported scales for use in their country, as definitions of quality of life are affected by national culture patterns [19, 20].

The study objective was to test the validity and reliability of the ASCQ-Me [13, 14] for use in the UK. This measure was chosen in preference to the SIMS, which consists of 142 items and was therefore considered too long to be practical by both clinicians and patients [12].

## Methods

### Study design

This was a cross-sectional study conducted at four National Health Service (NHS) hospitals in London.

### Population

Patients were invited to take part if they were adults aged ≥18 years, had a diagnosis of Sickle Cell Anaemia HbSS, Sickle C Disease HbSC or Sickle Beta Thalassæmia (HbSβThal) and their haematologist considered them well enough to answer the ASCQ-Me (either assisted or unassisted) [21].

### Data collection

Eligible patients were advised about the study by their haematologist when they attended a routine outpatient clinic appointment or hospital day care unit. Once consented, participants were given a copy of the questionnaire which they could complete in clinic or take home and return in a postage-paid envelope.

### Measures

The questionnaire pack included:

- ASCQ-Me Short Form [14]. A 30-item measure with 7 item banks: Pain episode frequency (2 items); Pain episode severity (3 items); Pain impact (5 items); Emotional impact (5 items); Social Functioning impact (5 items); Stiffness impact (5 items); and Sleep impact (5 items). The latter five item banks are each scored from 5 (never) to 1 (always). Scores on each subscale are standardised to have a mean of 50 and a standard deviation of 10. A higher score represents better HRQoL on all item banks, apart from pain episode frequency and severity, on which higher scores indicate greater frequency/severity. In the US, the ASCQ-Me has been shown to have excellent internal consistency for each item bank (≥.90) and the item banks differed significantly between SCD severity levels [16].
- The Medical Outcomes Study Short Form 36 (SF-36) [7] to assess generic HRQoL. The SF-36 is a 36-item measure with eight subscales: physical function; role limitation caused by physical function; pain; general health; energy/vitality; social function; role limitation caused by emotional difficulties; mental health. It also provides two composite scores for physical (PCS) and mental (MCS) HRQoL. Scores are transformed to a 0–100 scale on which the population mean is 50 and the standard deviation is 10. A higher score signifies better HRQoL. The SF-36 has previously shown to have good reliability and validity in the SCD population [22].
- Anxiety and depression were assessed with the Hospital Anxiety and Depression Scale (HADS) [23]. The HADS is a 14-item measure with individual scales for anxiety and depression. Each scale is scored from 0 to 21 with a higher score signifying greater anxiety or depression. A score of ≥8 indicates possible clinical depression/anxiety and a score of ≥11 indicates probable clinical depression/anxiety. The HADS has previously been validated in a clinical population [24], and been utilised in the UK SCD population [25].
- Symptoms: current pain, stiffness and fatigue were assessed with 10-point Visual Numeric Scales (VNS). Scores ranged from 0 to 10, with the higher scores indicating more pain, stiffness or fatigue [26].
- Number of days of college/work missed over the past month due to SCD, if applicable
- Current exercise tolerance: good, moderately reduced or severely reduced
- Number of painful crises managed at home during an average month/3 month period over the past 2 years

Routinely collected clinical data were extracted from participants’ medical notes with their informed consent. This included:

- Genotype (HbSS, HbSC or HbSβThal)
- Number of hospital admissions with pain crisis during the past 2 years
- Acute chest syndrome: Number requiring transfusion over the past 2 years
- History of:
  - Avascular necrosis (AVN) of hip
  - Stroke or recurrent transient ischemic attack (TIA)
  - Priapism
  - Severe, renal impairment: Requiring renal replacement treatment
  - History of Retinopathy with visual impairment
  - Elevated tricuspid regurgitation (TR) jet velocity
  - Catheter diagnosis of pulmonary hypertension
  - Recurrent ankle ulceration during past 2 years
  - Chronic pain: Persistent pain most days lasting more than 6 months
- Current medication with regular transfusion, medication with hydroxycarbamide, number of days of oral opioids used per week

Disease severity was classified based on the following criteria:

- People who have had ≥3 hospital admissions on average in the past 12 months vs those who have had < 3 admissions on average.

These disease severity criteria are the standard for pain episodes and have previously been used as entry criteria for a trial of hydroxyurea [27].

### Analysis

Study data were analysed using IBM SPSS Statistics 23®. The significance level was set at *p* < 0.01 in order to minimise the risk of a type I error. The pattern of missing data was evaluated using the missing data function. Any participant with more than 50% missing data was removed from the analysis. Little’s Missing Completely At Random (MCAR) test was conducted to check if there were any systematic differences between the missing values and the observed values [28]. All analyses were carried out as instructed by a predetermined statistical analysis plan that detailed all planned analyses prior to data collection.

### Reliability

Internal consistency was measured using Cronbach’s alpha coefficient to test the degree to which items in each ASCQ-Me subscale were related to each other. There are no tests of statistical significance for these estimations, though alphas > 0.70 are generally considered acceptable for aggregate data, with ≥0.80 to < 0.90 indicating good consistency, and > 0.90 excellent consistency [29].

### Validity

Content validity, defined as the extent to which the instrument measures the concept of interest, was confirmed prior to data collection by obtaining the views of patients with SCD and experts working in SCD on the questionnaire items to ensure that they capture the different components of SCD HRQoL. Construct validity, defined as evidence that the relationships among items conform to a priori hypotheses, was tested by examining convergent and known groups validity [30]. Convergent validity assesses measures that have an expected logical relationship with each other. This was tested by comparing the ASCQ-Me with the SF-36, HADS, and self-reported symptoms using Pearson’s correlations. For correlation of convergent validity, Pearson’s r values of < 0.20 are considered a very weak correlation, ≥0.20 to < 0.40 a weak correlation, ≥0.40 to < 0.60 moderate, ≥0.60 to < 0.80 strong, and ≥0.80 a very strong correlation [31]. Known groups validity assesses the extent to which measures are able to distinguish differences and similarities between sub samples, this was tested by comparing groups expected to differ on ASCQ-Me subscales using independent sample t-tests or analysis of variance (ANOVA).

Scores were compared between:

- People with different types of SCD - HbSS, HbSC, and HbSβThal.
- People who have an average of ≥3 hospital admissions per year over the past 2 years vs < 3 hospital admissions

Confirmatory factor analysis (CFA) of the ASCQ-Me was performed to examine the validity of the 5-factor structure.

The fit of the CFA model was assessed with comparative fit index (CFI), and root mean square error of approximation (RMSEA). CFI greater than 0.90 was considered an acceptable fit, and RMSEA < 0.07. RMSEA and CFI are standard statistical tests in CFA that assess the goodness of fit, this assesses how well the model-implied relationships of the items and the item banks are equivalent to the relationships in the sample data [32]. CFA was run using IBM® SPSS Statistics 23® AMOS 25.0. The standardized regression weights outputted in the CFA (Table 4) allow us to compare the means of individual items to the mean of each item bank in order to assess accuracy of fit of each item bank.

Further details of the analysis are included in the online Additional file 1: Table S1 and Table S2.

## Results

### Socio-Demographic & Clinical Details

A total of 224 patients consented to the study, of which 173 (77.2%) completed and returned the questionnaire. Sociodemographic details of the sample analysed are shown in Table 1. The sample had an average age of 36 years (range 18–78 years), were mostly female (57.8%) and the vast majority indicated that they were either black or black British (93.0%).

**Table 1:** Patient Socio-Demographic Characteristics (*n* = 173)

Variable	
Age years, mean (SD)	36.1 (12.5)
Gender, *n* (%)	
Female	100 (57.8)
Male	69 (39.9)
Undisclosed	4 (2.3)
Ethnicity, *n* (%)	
Black or Black British – African	118 (68.2)
Black or Black British – Caribbean	39 (22.5)
Black or Black British – Other	4 (2.3)
White and Black African	3 (1.7)
Other Mixed	1 (0.6)
White British	1 (0.6)
Undisclosed	7 (4.0)
Employment, *n* (%)	
Full time work	64 (37.0)
Unemployed	42 (24.3)
Part time work	24 (13.9)
Other	16 (9.2)
Student	15 (8.7)
Full time homemaker	6 (3.5)
Undisclosed	6 (3.5)
Highest Educational Qualification, *n* (%)	
Degree / Equivalent	70 (40.5)
A Level / Equivalent	36 (20.8)
Post graduate	34 (19.7)
GSCE / O level / Equivalent	25 (14.5)
No formal qualifications	4 (2.3)
Undisclosed	4 (2.3)

The clinical characteristics of the sample are reported in Table 2. Patients were mostly HbSS (72.3%), 20.8% with a HbSC diagnosis and a smaller number HbSβThal (4.0%). The number of transfusions used to treat acute chest syndrome over the previous 2 years was 0.7 (2.3). The results showed that the majority of participants had at least one pain crises per month on average (72.8%).

**Table 2:** Clinical Characteristics (*n* = 173)

Variable	
SCD Diagnosis, *n* (%)	
HbSS	125 (72.3)
HbSC	36 (20.8)
HbSβThal	7 (4.0)
Missing	5 (2.9)
Medical History, *n* (%)	
Avascular necrosis (AVN) of hip	39 (22.5)
Stroke or recurrent TIA	19 (11.0)
Priapism^a^	19 (26.0)
Severe, renal impairment: Requiring renal replacement treatment	11 (6.4)
History of Retinopathy with visual impairment	37 (21.4)
Elevated tricuspid regurgitation (TR) jet velocity	10 (5.8)
Catheter diagnosis of pulmonary hypertension	5 (2.9)
Recurrent ankle ulceration during past 2 years	14 (8.1)
Chronic Pain: Persistent pain on most days lasting more than 6 months	52 (30.1)
Acute Chest Syndrome – Transfusion required during the past 2 years, mean (SD)	0.7 (2.3)
Pain Crises, *n* (%)	
Had 3 or more hospital admissions with pain crises in the previous 12 months	13 (7.5)

^a^Percentage frequency calculated with male sample only

Scores on the SF-36 indicated impaired HRQoL; the physical composite score was more than one standard deviation (SD) below the standardised norm and the mental composite score was half a SD below. In reviewing the ASCQ-Me standardised scores for each item bank, the worst quality of life scores were seen in the social functioning item bank with a mean (SD) of 14.43 (5.22), followed by sleep 15.49 (4.67). Although HADS mean scores were in the normal range, HADS scores show that 46% of participants scored above the level for possible clinical anxiety and 41% for possible depression (Table 3). For 88% of the sample, their last pain attack had interfered with some aspect of their life and for 47% had lasted for 4 days or more.

**Table 3:** Patient Reported Descriptive Data

Variable	
Current Pain VNS, mean (SD)	2.8 (2.9)
Current Stiffness VNS, mean (SD)	2.5 (2.8)
Current Fatigue VNS, mean (SD)	4.0 (3.0)
HADS Anxiety, mean (SD)	7.6 (4.4)
HADS Anxiety Classifications, *n* (%)	
Non-cases (score of 0 to 7)	90 (54%)
Possible cases (score of 8 to 10)	35 (21%)
Probable cases (score of 11 to 21)	43 (25%)
HADS Depression, mean (SD)	7.1 (4.06)
HADS Depression Classifications, *n* (%)	
Non-cases (score of 0 to 7)	97 (59%)
Possible cases (score of 8 to 10)	46 (28%)
Probable cases (score of 11 to 21)	21 (13%)
SF-36 Physical Component Summary, mean (SD)^a^	37.26 (10.93)
SF-36 Mental Component Summary, mean (SD)^a^	44.02 (12.16)
ASCQ-Me: In the past 12 months, how many sickle cell pain attacks (crises) did you have? *n* (%)	
I did not have a pain attack	20 (11.6)
0	11 (6.4)
1	20 (11.6)
2	22 (12.7)
3	21 (12.1)
4 or more	79 (45.7)
ASCQ-Me: When was your last pain attack?, *n* (%)	
I’ve never had a pain episode	6 (3.5)
I have one right now	6 (3.5)
Less than a week ago	22 (12.7)
1–4 weeks ago	9 (5.2)
1–6 months ago	33 (19.1)
7–11 months ago	45 (26.0)
1–5 years ago	26 (15.0)
More than 5 years ago	26 (15.0)
ASCQ-Me Patient rating of pain severity in last attack (0 to 10), mean (SD)	7.17 (0.17)
ASCQ-Me: How much did your last pain attack (crisis) interfere with your life? *n* (%)	
I’ve never had a pain attack (crisis)	3 (2)
Not at all, I did everything I usually do	17 (10)
I had to cut down on some things I usually do	44 (25)
I could not do most things I usually do	41 (24)
I could not take care of myself and needed some help from family or friends	40 (23)
I could not take care of myself and needed constant care from family, friends, doctors, or nurses	28 (16)
ASCQ-Me: About how long did your most recent pain attack (crisis) last?	
I’ve never had a pain attack (crisis)	4 (2)
Less than 1 hour	8 (5)
1–12 hours	25 (15)
13–23 hours	7 (4)
1–3 days	47 (27)
4–6 days	38 (22)
1–2 weeks	28 (16)
More than 2 weeks	16 (9)
ASCQ-Me Pain Item Bank (7 day recall), mean (SD)	
How often did you have pain so bad that you could not do anything for a whole day?	3.27 (1.18)
How often did you have pain so bad that you could not get out of bed?	3.49 (1.20)
How often did you have very severe pain?	3.32 (1.17)
How often did you have pain so bad that you had to stop what you were doing?	3.18 (1.19)
How often did you have pain so bad that it was hard to finish what you were doing?	3.21 (1.18)
ASCQ-Me Sleep Item Bank (7 day recall), mean (SD)	
How often did you stay up most of the night because you could not fall asleep?	3.00 (1.89)
How often was it very easy for you to fall asleep?^a^	2.79 (1.13)
How often did you have a lot of trouble falling asleep?	3.06 (1.21)
How often did you stay up all night because you could not fall asleep?	3.39 (1.17)
How often did you stay up half the night because you could not fall asleep?	3.16 (1.11)
ASCQ-Me Stiffness Item Bank (7 day recall), mean (SD)	
How often were your joints very stiff when you woke up?	3.11 (1.29)
How often were your joints very stiff during the day?	3.29 (1.21)
How often were your joints so stiff during the day that you could not move?	3.88 (1.03)
How often did you wake up so stiff that you could not move?	3.89 (1.17)
How often did it take you a very long time to get out of bed because of stiffness?	3.69 (1.20)
ASCQ-Me Emotional Distress Item Bank (7 day recall), mean (SD)	
How often did you feel completely hopeless because of your health?	3.27 (1.39)
How lonely did you feel because of your health problems?	3.31 (1.38)
How depressed were you about your health problems?	3.40 (1.40)
How much do you worry about getting sick?	2.71 (1.39)
How often were you very worried about needing to go to the hospital?	3.04 (1.42)
ASCQ-Me Social Functioning Item Bank (30 day recall), mean (SD)	
How much did you rely on others to take care of you because of your health?	3.24 (1.17)
How often did your health slow you down?	2.65 (1.21)
How often did your health make it hard for you to do things?	2.74 (1.14)
How often did your health keep you from going out?	2.92 (1.16)
How much did your health make it hard for you to do things with your friends?	2.87 (1.20)
Standardised Total ASCQ-Me Item Bank, mean (SD)	
Pain	47.20 (10.05)
Sleep	50.04 (7.86)
Stiffness	49.22 (9.67)
Emotional Distress	46.63 (10.39)
Social Functioning	46.44 (10.15)

^a^Reverse scored

### Reliability

Cronbach’s alpha for the five primary ASCQ-Me item banks indicated that the sleep impact item bank had acceptable consistency (0.78), with the remaining 4 item banks showing excellent consistency (0.92–0.96).

### Construct validity

The CFA (Table 4) was assessed with the model fit indices comparative fit index (CFI), and root mean square error of approximation (RMSEA). All but one of the items loaded to their item respective bank (shown by a standardised regression weight of > 0.40). The CFI met the minimum criteria for acceptable fit at 0.94, the RMSEA marginally exceeded the threshold of < 0.07 at 0.08. The item “How often was it very easy for you to fall asleep?” did not load to the Sleep factor (< 0.40), therefore it was removed from the model.

**Table 4:** Confirmatory Factor Analysis of the ASCQ-Me

Item	Standardised Regression Weight^a^
Pain	
How often did you have pain so bad that you could not do anything for a whole day?	0.91
How often did you have pain so bad that you could not get out of bed?	0.88
How often did you have very severe pain?	0.91
How often did you have pain so bad that you had to stop what you were doing?	0.92
How often did you have pain so bad that it was hard to finish what you were doing?	0.95
Sleep	
How often did you stay up most of the night because you could not fall asleep?	0.57
How often was it very easy for you to fall asleep?	< 0.40
How often did you have a lot of trouble falling asleep?	0.87
How often did you stay up all night because you could not fall asleep?	0.90
How often did you stay up half the night because you could not fall asleep?	0.94
Stiffness	
How often were your joints very stiff when you woke up?	0.84
How often were your joints very stiff during the day?	0.80
How often were your joints so stiff during the day that you could not move?	0.87
How often did you wake up so stiff that you could not move?	0.90
How often did it take you a very long time to get out of bed because of stiffness?	0.90
Emotional Distress	
How often did you did you feel completely hopeless because of your health?	0.90
How lonely did you feel because of your health problems?	0.90
How depressed were you about your health problems?	0.86
How much do you worry about getting sick?	0.76
How often were you very worried about needing to go to the hospital?	0.76
Social Functioning	
How much did you rely on others to take care of you because of your health?	0.76
How often did your health slow you down?	0.93
How often did your health make it hard for you to do things?	0.93
How often did your health keep you from going out?	0.83
How much did your health make it hard for you to do things with your friends?	0.84

^a^The regression weight can be interpreted as the correlation between each item and its respective item bank

### Convergent validity

All correlations between ASCQ-Me item banks and the SF-36 and HADS were shown to be statistically significant (*p* < 0.01). For the HADS (Table 5), as expected, there was a strong relationship between the HADS anxiety and depression scales, and the emotional (Anxiety: *r* = − 0.66, Depression: *r* = − 0.64), and social impact ASCQ-Me banks (Anxiety: *r* = − 0.55, Depression: *r* = − 0.58). In reviewing the correlation between the ASCQ-Me item banks and SF-36 components, overall there were a number of moderate relationships. There was a stronger relationship between the ASCQ-Me pain item bank and the SF-36 physical component score (PCS, *r* = 0.52), than between the pain item bank and the SF-36 mental component score (MCS, *r* = 0.37). The emotional impact and social impact item banks of the ASCQ-Me had the strongest correlations with the mental component score of the SF-36 (Emotional: *r* = − 0.68, Social: *r* = − 0.61).

**Table 5:** ASCQ-Me Convergent Validity: Correlations with SF-36 & HADS

		Pain	Sleep Impact	Stiffness	Emotional Impact	Social Impact	Pain Crisis Frequency	Pain Crisis Severity
SF-36 Physical Component Score	r	0.52*	0.51*	0.51*	0.48*	0.65*	− 0.58*	-0.39*
	n	160	164	168	165	169	171	170
SF-36 Mental Component Score	r	0.37*	0.45*	0.37*	0.67*	0.61*	− 0.35*	− 0.34*
	n	160	164	168	165	169	171	170
HADS Anxiety	r	−0.24*	− 0.37*	− 0.35*	− 0.65*	− 0.54*	0.31*	0.23*
	n	160	163	165	163	167	168	167
HADS Depression	r	−0.36*	− 0.35*	− 0.34*	− 0.63*	− 0.58*	0.35*	0.27*
	n	154	158	161	159	162	164	163

*Significant result (*p* < .01)

### Known groups validity

There were no significant differences (*p* > 0.01) between patients with HbSS, Hb vhSC, or HbSßThal on any of the items banks (Table 6). Independent sample t-tests showed that all five of the ASCQ-Me item banks were able to significantly discriminate between a group of SCD patients that were admitted to the hospital three times or more on average in the previous 12 months compared to those that had been admitted twice or less (*p* < 0.01).

**Table 6:** ASCQ-Me Known Groups Validity

	SCD Diagnosis (ANOVA)				Hospital Admissions (T-test)		
	HbSS	HbSC	Hb SβThal	F(df), p	≥3 Hospital admissions in the previous 12 months	< 3 Hospital admissions in the previous 12 months	t(df), p
Pain Impact	46.62	49.42	49.16	1.16 (2, 156), 0.316	39.42	48.09	2.79 (153), 0.006*
Stiffness Impact	48.81	50.29	52.67	0.78 (2, 162), 0.460	41.64	50.23	3.17 (161), 0.002*
Sleep Impact	49.63	51.21	50.57	0.56 (2, 159), 0.571	44.35	50.60	2.79 (157), 0.006*
Emotional Impact	45.95	50.56	45.58	2.81 (2, 159), 0.63	36.15	47.83	4.11 (159), <0.001*
Social Impact	45.56	50.52	45.460	3.47 (2, 164), 0.34	39.58	39.58	4.193 (19), 0.001*

*Significant result (*p* < .01)

In exploratory analysis of the previous medical history of the SCD patients it was found that there were significant differences on all of the ASCQ-Me item banks between patients who had a history of persistent pain most days lasting more than 6 months and those who did not (*p* < 0.001) (Table S1). Furthermore, when reviewing medical history of avascular necrosis of hip (AVN), there were significant results for the stiffness ASCQ-Me item bank (*p* < 0.01), with patient that have had a history of AVN having lower scores indicating that they experience greater stiffness impact. There were no other significant results for any of the medical history items.

## Discussion

The results and analyses in this study show strong evidence of validity and reliability for the ASCQ-Me to be used as a measure of disease-specific HRQoL in adults with SCD in the UK. All of the item banks had good internal consistency, with the majority being excellent, after removal of one sleep item. The CFA indicated that the conceptual framework of the item banks fitted well for each item, and with the US ASCQ-Me [16]. The RMSEA test of model fit did not meet the minimum threshold for acceptance, however these values were shown to be akin to other self-reported questionnaires with a similar number of items [33], and similar to that seen in Keller et al. [16].

In reviewing the validity of the ASCQ-Me, the scale was compared with a generic QoL measure the SF-36, and the HADS. All ASCQ-Me item banks correlated significantly with the SF-36 subscales. As would be expected, the emotional impact item bank of the ASCQ-Me correlated more strongly with the SF-36 mental composite score than with the physical composite score, whereas the pain, stiffness and pain crisis frequency item banks correlated more strongly with the physical than the mental composite score. Sleep impact, social impact and pain crisis severity also correlated more strongly with the SF-36 physical than mental composite score, but only marginally. Anxiety and depression were strongly correlated with the emotional and social item banks of the ACSQ-Me, but weaker with the pain, sleep, and stiffness items. Although there is only a weak relationship between some item banks and the HADS, the stronger relationship between the HADS and the emotional ACSQ-Me item bank is to be expected, due to the HADS probing patients on the emotions surrounding depression and anxiety. We can therefore argue that the ASCQ-Me has convergent validity.

In reviewing the known-groups validity data, the ASCQ-Me did not distinguish between SCD diagnoses, supporting previous work using generic HRQoL measures [22, 34, 35]. This does however, contrast with the systematic review of HRQoL in SCD by Panepinto and Bonner [6] that did report a difference between genotypes, however they did not provide specific details on the differences or of which study/ies reported this finding, making any further interpretation difficult. It should be noted however, that there were only seven participants of the sample with HbSβThal, indicating less reliability of the diagnoses known groups validity test. However, it is not uncommon for clinical indicators to not predict HRQoL; the relationship between disease severity in long-term conditions and HRQoL is not always a linear one [36]. Keller [17] stated that SCD genotypes, due to the broad variation of symptomatology, are an unreliable indicator of disease severity. However, previous literature has suggested HBSS have more severe symptoms [37–39]. The current study found that HBSS had the poorest HRQoL on the ASCQ-Me item banks in comparison to the other genotypes, although this was not statistically significant.

The ASCQ-Me was able to successfully distinguish between groups of patients that were frequently admitted to hospital compared to those that were not. As would be expected, the results showed that patients who were admitted to hospital more had poorer quality of life in all ASCQ-Me items banks. Poorer scores on the SF-36 physical component summary were associated with a greater number of visits to the emergency department in one study [40] but another found no relationship between SF-36 scores and hospital service use or general practitioner visits [4]. The reason for these inconsistencies is most likely due to a number of factors, including clinical and socio-demographic differences between the samples, and a lack of reliable, valid, and consistent measurements of healthcare utilisation.

When compared to the ASCQ-Me field-test participants in Keller et al. [16], our sample reported more pain crises during the past 12 months, but the duration of participants’ most recent crisis, the percentage reporting that their last pain crisis interfered with their life and the level of pain severity experienced during the last pain crisis were very similar in the two samples. The current study also found that HRQoL in adults with SCD was impaired in relation to the general population, which confirms the findings of other research in this area. Anie, Steptoe, et al. [4] used the SF-36 and found that HRQoL was significantly lower than that of the UK general population. Pain, and the use of affective coping strategies, defined as catastrophizing, anger and fearful self-statements, praying and hoping, and isolation, were associated with poorer HRQoL. The ASCQ-Me provides further insight into HRQoL specific to SCD that generic measures such as the SF-36 fail to measure. It was seen that sleep impact and stiffness were of importance to quality of life as they both correlated with the SF-36 PCS and MCS, and the HADS anxiety and depression scales. This shows the importance of using a disease-specific measure such as the ASCQ-Me to assess HRQoL in SCD patients.

Using the ASCQ-Me in clinical practice could provide useful information to healthcare providers. The tool is easy for patients to complete and for clinicians to interpret. It could be used to obtain reliable assessments at each clinic visit of several important issues for people with SCD including stiffness, sleep, pain, emotional, and social impact of SCD. Not all of these factors are routinely assessed however this study has shown that they are negatively associated with the physical and mental well-being and therefore merit further attention in the clinical setting.

This study had a number of limitations. It could be argued that the SCD population was not representative of the UK general population as recruitment was only in London. This is also a limitation of other research that has examined HRQoL in people with SCD in the UK [4]. However, approximately two-thirds of people with SCD in the UK live in London with most others living in other large urban areas [41]. Although the analysis shows relationships between the measured variables, due to the limitations of correlation analysis, cause cannot be inferred. As some data were extracted from patients’ medical notes, we acknowledge that there may be some inconsistencies in these data; this is an issue for all studies that extract data from medical notes.

Future studies could be carried out to provide further reliability and validity for the ASCQ-Me scale, this includes test-retest reliability. By measuring changes in severity mapped against changes in ASCQ-Me scores over time any clinical responsiveness of the scale would show further validity of the questionnaire. Such reliabiltiy and validity tests have so far not been carried out on the ASCQ-Me US version.

## Conclusion

The analyses show strong evidence of reliability and validity for the ASCQ-Me to be used as a measure of disease-specific HRQoL in SCD in the UK, replicating some of the findings of the US ACSQ-Me. The UK measure will be a valuable tool for assessing the HRQoL of adults with SCD, providing a useful outcome measure in both research and clinical practice.

## Additional file

Additional file 1: **Table S1.** ASCQ-Me Medical History Differences. **Table S2.** Current treatment. Descriptive statistics for treatments taken by two or more participants (DOCX 23 kb)

## Abbreviations

ASCQ-Me: Adult Sickle Cell Quality of Life Measurement Information System
AIMS: Arthritis Impact Measurement Scale
ANOVA: Analysis of variance
AVN: Avascular necrosis
CFA: Confirmatory factor analysis
CFI: Comparative fit index
EQ-5D: EuroQol five-dimensional questionnaire
HADS: Hospital Anxiety & Depression Scale
HbSC: Haemoglobin SC
HbSS: Haemoglobin SS
HRQoL: Health-related quality of life
MCAR: Missing Completely At Random
MCS: Mental component score
NRES: National Research Ethics Service
PCS: Physical component score
RMSEA: Root mean square error of approximation
SCD: Sickle cell disease
SF-36: RAND Medical Outcome Survey Short Form 36
SIMS: Sickle Cell Impact Measurement Scale
HbSβThal: Haemoglobin Sβ-thalassæmia Hb
TIA: Transient ischemic attack
TR: tricuspid regurgitation
VNS: Visual numeric scales
