# Supplementary material for: Psychometric analysis of the adult sickle cell quality of life measurement information system (ACSQ-Me) in a UK population
Source: Health Qual Life Outcomes. 2019 Apr 29;17:74. doi: 10.1186/s12955-019-1136-7 (PMC6489218; doi:10.1186/s12955-019-1136-7)
Supplement: Supplementary file 1 — Table S1. ASCQ-Me Medical History Differences. Table S2. Current treatment. Descriptive statistics for treatments taken by two or more participants (DOCX 23 kb) [file 12955_2019_1136_MOESM1_ESM.docx]

## Table S1. ASCQ-Me Medical History Differences

|  | **Medical History - Avascular Necrosis of Hip** | | | **Medical History - Chronic pain: Persistent Pain Most Days Lasting More than 6 months** | | | **Stroke or recurrent transient ischemic attack** | | |
| --- | --- | --- | --- | --- | --- | --- | --- | --- | --- |
|  | **No** | **Yes** | **t(df), p** | **No** | **Yes** | **t(df), p** | **No** | **Yes** | **t(df), p** |
| **Pain Impact** | 48.31 | 43.94 | 2.23 (157), 0.023 | 49.78 | 41.55 | 5.66 (113), <0.001* | 47.56 | 45.84 | 0.643 (156), 0.521 |
| **Stiffness Impact** | 50.54 | 45.49 | 3.33 (9), 0.001* | 51.85 | 43.87 | 5.37 (165), <0.001* | 49.74 | 46.55 | 1.359 (164), 0.176 |
| **Sleep Impact** | 50.64 | 47.84 | 1.62 (47), 0.112 | 51.85 | 45.78 | 4.82 (161), <0.001* | 49.99 | 50.44 | -0.231 (160), 0.818 |
| **Emotional Impact** | 47.52 | 44.60 | 1.54 (162), 0.125 | 49.15 | 41.75 | 4.53 (162), <0.001* | 47.29 | 44.12 | 1.246 (161), 0.214 |
| **Social Impact** | 47.48 | 43.64 | 2.09 (166), 0.038 | 49.13 | 40.92 | 5.91 (134), <0.001* | 46.99 | 44.44 | 1.015 (165), 0.311 |
|  | **Priapism** | | | **Severe, renal impairment: Requiring renal replacement treatment** | | | **History of Retinopathy with visual impairment** | | |
|  | **No** | **Yes** | **t(df), p** | **No** | **Yes** | **t(df), p** | **No** | **Yes** | **t(df), p** |
| **Pain Impact** | 49.77 | 44.33 | 1.952 (66), 0.055 | 47.66 | 43.14 | 1.442 (157), 0.151 | 47.83 | 45.34 | 1.205 (157), 0.230 |
| **Stiffness Impact** | 51.21 | 45.93 | 2.012 (69), 0.048 | 49.78 | 43.45 | 2.137 (165), 0.034 | 50.05 | 46.96 | 2.039 (76), 0.045 |
| **Sleep Impact** | 52.04 | 49.58 | 1.182 (66), 0.242 | 50.19 | 47.40 | 1.088 (161), 0.278 | 50.22 | 49.24 | 0.634 (161), 0.527 |
| **Emotional Impact** | 49.23 | 46.58 | 0.955 (68), 0.343 | 47.21 | 41.75 | 1.720 (162), 0.087 | 47.09 | 45.98 | 0.573 (162), 0.567 |
| **Social Impact** | 49.48 | 45.40 | 1.416 (69), 0.161 | 46.99 | 40.84 | 1.964 (166), 0.051 | 47.01 | 45.09 | 1.017 (166), 0.311 |

*Significant result (p<.01)

Table S1 (continued). ASCQ-Me Medical History Differences

|  | **Catheter diagnosis of pulmonary hypertension** | | | **Recurrent ankle ulceration during past 2 years** | | | **Elevated tricuspid regurgitation (TR) jet velocity** | | |
| --- | --- | --- | --- | --- | --- | --- | --- | --- | --- |
|  | **No** | **Yes** | **t(df), p** | **No** | **Yes** | **t(df), p** | **No** | **Yes** | **t(df), p** |
| **Pain Impact** | 47.22 | 55.80 | -1.69 (156), 0.092 | 47.60 | 44.78 | 1.000 (157), 0.319 | 47.41 | 44.40 | 0.871 (155), 0.385 |
| **Stiffness Impact** | 49.49 | 48.84 | 0.150 (164), 0.881 | 49.86 | 43.95 | 2.229 (165), 0.027 | 49.40 | 47.13 | 1.280 (14), 0.222 |
| **Sleep Impact** | 50.07 | 48.36 | 0.478 (161), 0.633 | 49.96 | 50.61 | -0.292 (161), 0.770 | 50.02 | 49.58 | 0.169 (159), 0.866 |
| **Emotional Impact** | 47.10 | 42.90 | 0.909 (161), 0.365 | 46.93 | 45.89 | 0.362 (162), 0.718 | 47.00 | 42.64 | 1.313 (160), 0.191 |
| **Social Impact** | 46.86 | 41.92 | 1.084 (165), 0.280 | 46.78 | 44.55 | 0.786 (166), 0.433 | 46.39 | 48.80 | -0.726 (164), 0.469 |

*Significant result (p<.01)

## Table S2. Current treatment. Descriptive statistics for treatments taken by two or more participants.

| **Treatment** | **Number of participants currently taking treatment** |
| --- | --- |
| Antibiotic | 137 |
| Folic acid | 129 |
| Strong opioid | 84 |
| Moderate strength opioid | 82 |
| Paracetamol | 57 |
| Non-steroidal anti-inflammatory | 46 |
| Hydroxycarbamide | 29 |
| Vitamin D | 28 |
| Lansoprazole | 11 |
| Iron chelation | 10 |
| Amlodipine | 7 |
| Anticoagulation | 6 |
| Cyclizine | 6 |
| Omeprazole | 6 |
| Amitriptyline | 5 |
| Aspirin | 5 |
| Ramipril | 5 |
| Senna | 5 |
| Hydroxycholoroquine | 4 |
| Calcium | 3 |
| Citalopram | 3 |
| Docusate sodium | 3 |
| Gabapentin | 3 |
| Phenoxymethylpenicillin | 3 |
| Prednisolone | 3 |
| Salbatamol | 3 |
| Alendronic acid | 2 |
| Azathiprine | 2 |
| Ceterizine tablets | 2 |
| Chlorophenamine | 2 |
| Etilefrine | 2 |
| Methotrexate | 2 |
| Perindopril | 2 |
| Pregablin | 2 |
| Propranolol | 2 |
| Seretide inhaler | 2 |
| Solpadol | 2 |
| Spironolactone | 2 |
| Sulfasalazine | 2 |
